# Acoustic Performance of Stone Mastic Asphalts with Crumb Rubber and Polymeric Additives in Warm, Dry Climates

**DOI:** 10.3390/ma19020260

**Published:** 2026-01-08

**Authors:** Jesús Campuzano-Ríos, Juan José Jorquera-Lucerga

**Affiliations:** Mining and Civil Engineering Department, Universidad Politécnica de Cartagena, 30202 Cartagena, Spain; juanjo.jorquera@upct.es

**Keywords:** traffic noise, noise reduction, tire-pavement interaction, Stone Mastic Asphalt, crumb rubber, polymeric additives, Mediterranean climate

## Abstract

Traffic noise is one of the main sources of environmental problems and a growing challenge for national traffic authorities. It is widely accepted that tire-pavement interaction is the main cause of traffic noise at speeds between 40 and 90 km/h. Typically, noise attenuation strategies include earthworks, tree belts, or noise barriers. However, a solution that is almost always viable is the use of low-noise pavements, which are characterized by their porous macrotexture, such as Stone Mastic Asphalt (SMA) mixtures. These mixtures are increasingly used for heavy traffic volumes because of their many advantages, including drainage properties and mechanical strength. Based on the experimental results obtained on different roads in southern Spain, this paper compares noise reduction in an SMA standard mixture due to the incorporation of different additives, such as crumb rubber and polymeric additives. According to the analysis, increasing the additives content by 1% reduces CPX by 1.18 decibels, approximately, and none of the analyzed sections shows increases greater than 3 dB within 24 months. Additionally, the paper proposes design recommendations regarding macrotexture and the percentage of voids for zones with warm, dry climates, such as Mediterranean Spain.

## 1. Introduction

**Noise and health.** The health impacts of environmental noise are a growing concern among both the general public and policy-makers in Europe. Environmental noise, also known as noise pollution, is among the most frequents sources of complaint regarding environmental issues in Europe, especially in densely populated urban areas and residential areas near highways, railways and airports. According to World Health Organization (WHO) [1], evidence exists on the relationship between environmental noise and specific health effects, including cardiovascular disease, cognitive impairment, sleep disturbance, tinnitus and annoyance. Even with conservative assumptions applied to the calculation methods, the results obtained by WHO [1] indicate that at least one million healthy life years are lost every year from traffic-related noise in the western part of Europe, where sleep disturbance and annoyance comprise the main burden of environmental noise.

Other numerous studies have shown that traffic noise has effects on cardiovascular health, sleep disorders, or declining cognitive performance in children (see, for example, respectively [2,3,4]). According to a recent meta-analysis [5], road traffic noise ranks among the four environmental risk factors with highest health impact in European countries, which means a loss of 400–1500 healthy life years due to ischemic heart disease per million people. The impact of urban noise on public health (8% of the environmental burden of disease) was rated medium-high, comparable to that of secondhand smoke and radon, and only behind fine particles (PM2.5). In Madrid, for example, the relationship of road traffic noise with cardiovascular, respiratory, and diabetes-related mortality [6] has been demonstrated.

In addition, wildlife species, at both the individual and population levels, are also affected by the anthropogenic noise sources introduced into the environment due to human development. The types of impacts range from chronic stress and associated physiological responses, damage to the auditory system, to the masking of sounds important for survival and reproduction, with a consequent decline in population size [7].

**Administration.** For these reasons, the EU is addressing environmental noise. The population’s exposure to noise levels is regulated in the European Union by European Directives, such as Directive 2002/49/EC [8] and Commission Directive (EU) 2015/996 [9], which do not set minimum values but do oblige member states to do so. In the case of Spain, Law 37/2003 [10] on Noise and its implementing regulation by the Royal Decree (RD) 1513/2005 [11] (Assessment and Management of Environmental Noise) transpose Directive 2002/49/EC [8] and require the preparation of Strategic Noise Maps and periodic Action Plans. In addition, RD 1367/2007 [12] sets Acoustic Quality Objectives with immission values for different types of areas: residential, for health/educational/cultural use, or for tertiary use. If the values exceed these limits, corrective measures must be taken to improve the acoustics of the area until the required levels are reached.

**Traffic noise.** Traffic noise is considered one of the main sources of environmental problems and a growing challenge for national traffic authorities. Traffic noise is the sum of the noise produced by each individual vehicle in a given traffic situation. At low speeds, such as those found on urban roads, most of the noise produced by an individual vehicle comes from air intakes, engines, fans, transmissions, exhaust pipes, brakes, and bodywork. Vehicles are generally quietest when traveling at around 30 km/h. Poor driving practices and stop-start conditions, such as those caused by congestion and traffic lights, increase noise levels [13]. For this reason, road infrastructure owners and managers have gradually paid more attention to the growing problem of traffic noise. The effect of noise is mentioned, for example, in the CEDR’s Action Plan 2025–2027 [14] as one of the main future challenges for road infrastructure.

**Noise generation.** According to Priede [15] and Vaitkus [16], the noise generated by a vehicle is due to three main sources: the propulsion (i.e., engine, transmission system and accessories), the aerodynamic noise and the road excitation due to the interaction between the tire and the pavement. The tire-pavement interaction noise (TPIN) is defined as the noise emitted from a rolling tire because of the interaction between the tire and the road surface [17], which is also known as tire-road interaction noise, tire/pavement noise, tire/road noise (TRN), or tire noise [18]. At lower speeds, up to 40 km/h, most of the overall noise is due to propulsion, while at higher speeds (40–100 km/h) TPIN prevails and contributes to approximately 90% of the acoustic energy generated.

Heckl [19] pioneered the analysis of the physical mechanisms of noise generation in road traffic by compiling the experimental evidence available at the time, and concluded that, above 50 km/h, the dominant source in passenger cars is tires. This result justified the shift in noise mitigation strategies towards treating the wheel-pavement interface (texture, porosity) in Europe, since, above 50–60 km/h, the dominant traffic noise is TPIN. Despite its pioneering nature, its conclusion has been confirmed by subsequent literature and by measurements of the noise produced at the source. Al-Masaeid et al. [20], in a much more recent study on urban roads with continuous data and modeling, conclude that, at actual average speeds, tire-road interaction is the main source for passenger cars above 40 km/h (and, by extension, above 50–60 km/h on higher speed roads), while at lower speeds the contributions of propulsion increase. Only at very high speeds, and not always, does aerodynamic noise start to be the main source of noise from the vehicle.

**Main corrective measures.** The main corrective measures to mitigate the impact of traffic noise can be combined to solve the same problem (see, for example, [21]). The first is, of course, to take action at the design stage: appropriate earthworks can take advantage of the noise reduction associated with embankment or cut roads (see, for example, [22]), or even covered roads, as in the case of the M-30 and Madrid Río tunnels in Madrid [23]. Noise reduction can also be significant through proper traffic planning and management, especially in noise-sensitive areas, which can achieve less traffic, smoother flow, and quieter conditions. Another approach is to attenuate noise that has already been produced by installing noise-attenuating elements such as vegetation belts adjacent to roads [24,25] or acoustic screens [26]. The third approach is to reduce noise produced at source by traffic, such as vehicle propulsion (e.g., by using electric vehicles [27] or vehicle-pavement interaction [19]). These strategies are described below.

However, especially in urban environments, where this problem is particularly severe, not all of these strategies are feasible: burying an existing road to avoid noise problems is a very costly solution; planting a vegetation belt generally requires too much room; and noise barriers, when possible, create visual obstacles, alter the landscape, and sometimes fail to achieve the desired effect.

**Low-noise pavements and SMA.** However, one solution that is almost always viable is the use of low-noise pavements, which reduce TPIN. Among these pavements, characterized by their porous macrotexture [28], are SMA (Stone Mastic Asphalt, also known as Stone Matrix Asphalt) mixtures. These types of mixtures are increasingly used [29] because they offer many advantages, such as their drainage properties and mechanical strength. Normally, SMA mixtures are primarily used on pavements carrying heavy traffic volumes or on pavements carrying heavy loads and/or high tire pressures [30]. The increase in service life and improved performance can offset the additional cost when using SMA in lieu of dense-graded mixtures. In addition, the durability of SMA is equal or greater than close-graded asphalt and significantly greater than open-graded asphalt. SMA is suitable for use at intersections and other traffic stress situations where open-graded asphalt is unsuitable [31], where these noise problems are also concentrated, such as roundabouts [32]. Among the studies conducted on these mixtures is the Soundless Project [33], carried out by the Regional Government of Andalusia in southern Spain, which has studied these asphalt mixtures using different waste materials as additives [34].

**Research objective.** Therefore, the objective of the research described in this paper is to collect data from Soundless and other projects and carry out a comparative study on the noise produced by different SMA mixtures with different additives, in order to analyze the noise reduction in each of them with respect to a standard mixture. In addition, the objective is to propose design recommendations for countries with a Mediterranean climate, such as Spain.

**Paper structure.** This paper is structured as follows: Section 2 describes the problem of traffic noise and the context of the study. Section 3 describes existing strategies for reducing traffic noise impact. Section 4 details the characteristics of SMA, their advantages and potential to reduce traffic noise, and their use as low-noise pavements. Section 5 compares several SMA with different additives to analyze the noise reduction achieved by each one. Finally, Section 6 analyzes and discusses the results of the study. The paper concludes with Section 7, which presents the main conclusions drawn from the study and design recommendations for hot and dry climates, such as Mediterranean.

## 2. Noise Generation and Traffic Noise Assessment

### 2.1. Noise Generation

**Figure 1:**
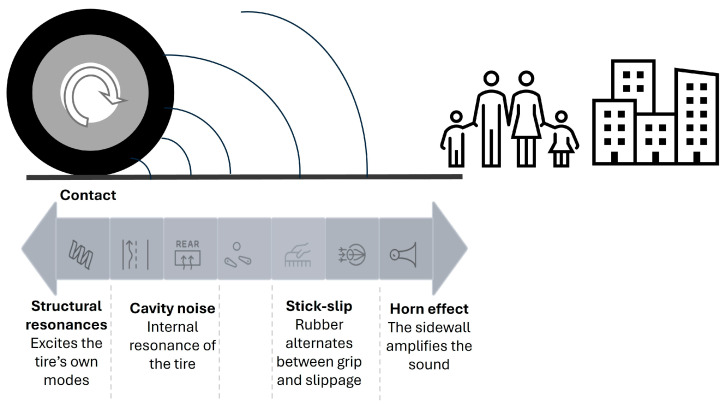
Noise generation due to tire-pavement interaction.

When a tire rolls on the road surface, mechanical and aerodynamic interactions [28] occur within a small area known as the ‘contact patch’. These interactions excite vibrations and air flows, which subsequently generate noise in the form of acoustic waves that propagate into the environment (See Figure 1). Above 50–60 km/h, this noise is usually louder than the engine noise [19]. The aerodynamic interactions are the following:

- Air pumping: the air trapped in the cavities of the texture and between the tire tread and the pavement is compressed and expelled violently, generating very significant acoustic pulses between 1 and 4 kHz. Winroth, J. et al. [35] support the recommendation of “negative” textures and connected voids to reduce the aerodynamic component.
- Horn effect: The geometry formed by the sidewall and the wearing surface acts as an amplifier of the noise generated in the contact zone, with increases of several dB [36].
- Tire cavity noise: the internal volume of the tire acts as a resonator and a peak usually appears around 180–220 Hz, depending on size and pressure. It is excited by impacts and irregularities and modulates the total spectrum [19].
- Wet pavement. The noise level increases by approximately 15 dB due to the presence of water [36]. In another study conducted in Portugal, Freitas et al. [37] measured an increase of 6–7.5 dB in passenger cars and 3–5 dB in heavy vehicles on consecutive sections of porous and dense pavement. In Cai et al. [38], also on wet road surfaces, the increase was 10 dB, 5–6 dB, and 4 dB for light, medium, and heavy vehicles, respectively. Even with sound-absorbing, draining, or porous asphalt (PA) pavement, the presence of water increases the TPIN. According to [39], the pavements ranked from highest to lowest noise reduction are: a draining pavement in dry conditions, a dense pavement in dry conditions, a wet or damp draining pavement, and, lastly, a wet dense pavement.

Mechanical interactions include:

- Vibration of the tread blocks: the tread blocks move in and out of the footprint, periodically loading and unloading and exciting vibrations that emit sound. The stiffness of the compound, the tread geometry and the macrotexture of the pavement determine the amplitude and frequency (typically 0.8–2 kHz for cars). Larsson et al. [40] model the dynamic behavior of tread blocks and their coupling with pavement roughness, enabling the design of textures that minimize block vibration.
- Stick-slip: at the microscale, rubber alternates between sticking to and slipping off the peaks of the texture. This phenomenon generates broadband vibrations, which increase with effective roughness and tangential force, for example during acceleration, braking and cornering [41].
- Roughness impact: when the pavement texture has megatexture (e.g., bumps or joints) or wavelengths comparable to the size of the studs, slower excitations (i.e., low frequencies) and spectrum modulations are created. Del Pizzo et al. [42] corroborate the idea that a well-designed macrotexture, together with moderate porosity, reduces mechanical and aerodynamic excitation.
- Tire structural resonances: [28] The tire rims, plies and sidewalls all have their own modes. When these are fed by texture excitation, specific bands of the sound spectrum increase.

### 2.2. Traffic Noise Assessment

In Europe, traffic noise assessment employs integrated indicators such as L_den_ (day, evening, night) and L_night_, in accordance with the European Environmental Noise Directive [8]. They represent the annual average noise level over a 24-h period, with a special weighting given to noise that occurs during the evening and night, as these times are considered more disruptive. These noise maps are used to evaluate the impact of noise on human health and well-being and are created to meet the requirements of the Environmental Noise Directive. As previously referenced in [4,5], at speeds above 40–60 km/h, the predominant component becomes rolling noise (TPIN). Consequently, the selection of the wearing course and its texture is pivotal in determining actual noise exposure.

At the project level, the characterization of pavement sound behavior is based on standardized methods: CPX (Close Proximity) to evaluate the contribution of the pavement and SPB (Statistical Pass By) to evaluate the overall effect on traffic flow in situ. Macrotexture is characterized by the Mean Profile Depth (MPD) and is functionally linked to noise generation and skidding.

The CPX procedure is a standardized method for measuring tire-road noise and evaluating road surfaces acoustically. The process involves a set of standardized reference tires that are identical for all measurements, ensuring consistency. Microphones are positioned at a fixed distance of 20 cm from the tire to capture the noise generated by the interaction between the tire and the road surface. The test is conducted at specific, constant speeds (e.g., 50, 80, or 100 km/h). The measurement setup is designed to isolate tire-road noise, often by using a trailer with an enclosed chamber to shield the microphones from external sounds such as traffic noise. The method involves the collection of data on sound pressure levels at various points along a road section. This data is averaged over specified distances in order to account for the road’s condition. Measured noise levels are corrected for factors such as ambient temperature and tire rubber hardness, thus ensuring they are comparable. More details can be found on [43].

The SPB procedure is a method for measuring road surface noise by analyzing the sound levels of vehicles passing a fixed point, as described in ISO 11819-1 [44]. The process involves measuring noise from various vehicle categories at different speeds, correcting the data for conditions like air temperature, and then calculating a single “SPB index” to compare the acoustic performance of different road surfaces. A lower SPB index indicates reduced noise generation. In order to measure the noise of light, medium-weight and heavy vehicles as they pass by, microphones are placed at a defined distance from the road. Key variables, such as wind speed, air temperature, and road surface temperature, are recorded during the measurements. The measured noise levels are corrected to reference conditions, typically a temperature of 20 °C. Once the data has been obtained, the noise levels are plotted against vehicle speed. Linear regression is used to determine the noise level for each vehicle category at a defined reference speed. The results for different vehicle categories are weighted and summed up to create the final SPB index. The SPB index provides a single value for the road surface’s noise performance, allowing for direct comparison with other surfaces. A lower index indicates a quieter road surface. More details can be found on [44].

Mean Profile Depth (MPD) is a measure of a pavement’s macrotexture, calculated from a 2D surface profile by averaging the mean segment depths over a specific length. It is derived by applying filters to the surface profile, dividing the baseline into segments, and then calculating the average depth of each segment’s peaks relative to its baseline, as described in [45].

## 3. Strategies for the Reduction in Traffic Noise Impact

The main strategies for reducing traffic noise impact are the following:

1. Careful planning and traffic management that separates traffic from noise-sensitive areas. According to Lay [13], noise problems can be avoided, mainly by planning strategies, zoning controls and building regulations, which means, respectively, adopting measures such as keeping traffic routes away from noise-sensitive land-uses; preventing noise-sensitive uses from being located near traffic routes, or requiring buildings in noise-sensitive uses to be appropriately located, designed and insulated. An alternative solution is introducing noise-tolerant land-uses, which may be expensive as it will usually involve purchasing noise-affected properties and selling them to new residents who are less concerned with the noise level. The at-source factor that can most reduce noise problems is traffic management since it can achieve less, slower and smoother traffic flow. Additionally, noise can be reduced with fewer noisy vehicles, particularly noisy trucks. For example, the objective of the EU Silence Project [46] is to advise city authorities on types and packages of traffic flow measures and driver assistance systems which can be used to reduce noise from road traffic.
2. Attenuating the impact of noise that has already been generated, using noise barriers that are placed between the source of the noise and the perceiver of the noise. Once noise has been generated, it can be reduced (i.e., attenuated) by noise barriers, which may be earth mounds, the faces of cuttings, crib walls, rock walls, concrete walls, or timber fences, as detailed below.
3. Reducing noise prior to its generation, i.e., minimizing noise generated at its source by acting on the vehicle’s propulsion system, aerodynamic noise, and TPIN.

### 3.1. Earthworks

**Figure 2:**
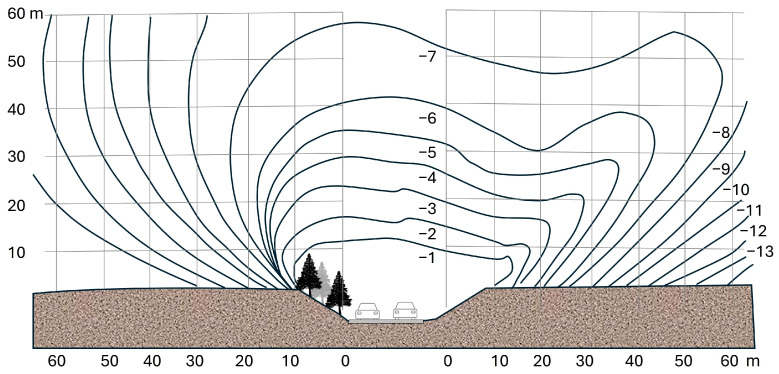
Noise reduction in a road in cutting. (**Left**): slope with vegetation. (**Right**): slope without vegetation (Redrawn from [22]).

Noise can be reduced during the design stage by adjusting the elevation of the road. Roads built on embankments and cuttings are quite effective at reducing noise compared to roads that remain at ground level. In cuttings, noise can be reduced by 5 to 10 dBA depending on the depth. The cutting slopes should be as steep as possible to ensure maximum effectiveness. In urban and built-up areas, this measure is costly and difficult to implement, requiring technical solutions to resolve water drainage issues. Costs of additional earthworks must also be considered [22]. However, to achieve a significant reduction in noise, it is necessary to build soft earth walls, preferably planted with shrubs and trees, so that they offer protection to neighboring populations. In rural areas, roads in cuttings are a more suitable solution from a cost-effectiveness perspective (Figure 2).

Roads built on embankments are also more effective from an acoustic point of view than those at ground level. The height of the embankment must exceed 2.5 m and the slope must be absorbent. This solution can also contribute to the balance of earthworks, in which the amount of excavation should be equal to the amount of fill. As in the case of roads in cuttings, this solution is more effective in rural areas than in urban areas.

### 3.2. Tree Belts

**Figure 3:**
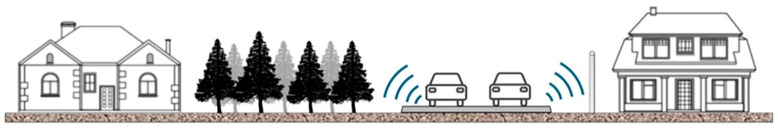
Noise reduction strategies. Left: Tree belts. Right: Noise barriers.

Vegetation has been proposed as a natural material for reducing acoustic energy outdoors [47]. The effect of different types of tree belts has been studied, for example, by Fang and Ling [25]. Vegetation can be used to reduce traffic noise by acting as a natural barrier that absorbs, deflects, and disperses sound waves (Figure 3). Although it does not eliminate noise completely, a well-designed vegetation barrier can significantly reduce noise pollution levels, especially high-frequency noise. Leaves and branches decompose and disperse sound, reducing its intensity. Plants with fleshy leaves and thick bark are particularly effective. Evergreen trees are preferable as they retain their properties throughout the year. In addition, vegetation-covered soil also absorbs noise, especially lower frequencies [47]. To minimize noise, the vegetation barrier should be dense and deep, at least 30 m wide, as tall as possible, and combine trees and shrubs. It is also advisable to place them as close as possible to the source of the noise or the receiver.

The main disadvantages are that noise absorption capacity varies depending on the time of year, especially with deciduous trees, and that its effectiveness depends greatly on the species, density, and distance from the noise source. In addition, vegetation is less effective at dampening low-frequency noises, such as those produced by heavy traffic. To create an effective vegetation barrier, a wide area with dense vegetation is required, making it a solution that is rarely possible in urban and peri-urban environments.

### 3.3. Noise Barriers

Earth mounds, constructed alongside major earthworks, are usually the most economical form of noise treatment as they often utilize material left over from pavement excavation. According to Lay [13], a typical noise barrier reduces noise levels by around 10 dB (i.e., halving the noise), rising to 20 dB if the noise path increases by 3 m or more. Barriers (Figure 3) should be located to at least prevent any line-of-sight between noise source and receiver. The problem is complicated in urban areas, since large, solid buildings can reflect or scatter sound in a complex manner. The barriers themselves usually reflect any sound that strikes them upwards and/or back across the road. Thus, absorptive barriers may be required if reflective barriers could worsen conditions on the opposite side of the road. Barriers require a mass of approximately 3 kg/m^2^ to prevent excessive transmitted sound.

The acoustic and non-acoustic performance of screens can be significantly reduced by exposure to the elements, splashes, UV radiation and maintenance agents, and therefore their sustained effectiveness requires periodic inspection and replacement [26]. In terms of network management and social acceptability, quiet pavements offer better integration (with no visual impact) and provide widespread benefits: they reduce sound power for all receivers, including those in areas where it would be impractical or ineffective to install screens [48]. Indeed, European programmers on noise-reducing pavements recommend prioritizing their evaluation as part of action plans and road maintenance, and restricting screens to scenarios where their use is justified by the geometry and territory occupation.

Screens are a localized measure to prevent noise propagation. They can be effective in specific scenarios where the topography is favorable, and receivers are nearby and at the same elevation. However, they have significant disadvantages: landscape intrusion and visual barriers; spatial fragmentation and its effects on wildlife and birds; shade and possible microclimatic effects; reflections and leaks at intersections, access points and viaducts, which reduce their overall effectiveness; limitations for receivers at high elevations (upper floors); and implementation and maintenance costs (e.g., cleaning, graffiti removal and material degradation) that compromise their performance throughout their life cycle.

These conclusions are consistent with European recommendations [49] which, while recognizing the usefulness of screens in some cases, recommend incorporating low-noise pavement into planning and maintenance as a primary mitigation measure: (a) CEDR Technical Report 2017-01: Noise-Reducing Pavements, a European summary containing implementation and management recommendations; (b) CEDR Technical Report 2017-02: Noise Barriers, showing that acoustic and non-acoustic deterioration of barriers over time has major maintenance implications and higher costs; (c) the FHWA/ADOT Quiet Pavement Pilot Program, which provides a life cycle assessment and analysis methodology for comparing strategies (quiet pavements vs. barriers).

As a main conclusion, reducing noise at the source (i.e., at the tire-road interface) offers direct, immediate and sustainable benefits [50], as well as providing an alternative to other management measures such as speed limits, traffic management or noise barriers, which should be reserved for cases with space limitations.

### 3.4. Actions on Vehicles

In its 1995 study, ‘Noise Reduction in the Road Environment’ [22], the Organization for Economic Co-operation and Development (OECD) indicated that reducing vehicle noise emissions by between 5 dB and 10 dB would cost around 2–5% of the price for light vehicles and 5–9% for heavy vehicles. Such additional costs imply significant investment by automotive companies and lengthy implementation periods. The emergence of electric vehicles does not currently significantly change the situation, as the same study established that wheel-road noise is 2–4 dB higher than other noises produced by light and heavy vehicles travelling above 50 km/h. However, as previously mentioned, tire-pavement noise dominates at medium-high speeds in all cases. Therefore, replacing combustion engines with electric ones would hardly change noise levels on highways and expressways unless action is also taken regarding the road surface, tires, and speed. Verheijen and Jabben [51] and Skov and Iversen [52] demonstrate that differences in noise generation between electric and combustion vehicles are primarily evident at low speeds on urban streets, where electric propulsion reduces noise by several dB.

All new hybrid electric and pure electric vehicles in the European Union (EU) [53] must be equipped with an Acoustic Vehicle Alerting System (AVAS) to warn pedestrians when travelling at low speeds. This creates a potential conflict between safety and nuisance. However, the AVAS breaks part of the ‘silence’ in pedestrian environments at low speeds (below 20 km/h) to improve road safety. Therefore, below ~20 km/h, the electric powertrain is not completely silent by design. Furthermore, the axle load and tires of electric vehicles are heavier than those of combustion vehicles. This increased load and tire width can increase rolling noise. For this very reason, Leupolz and Gauterin [54] prioritize tire-pavement optimization.

### 3.5. Low-Noise Pavements

Low-noise tires and road surfaces are two important solutions that have the potential to reduce noise levels on roads at the source and thus improve the quality of life for residents. For example, Licitra et al. [50], based on the Leopoldo project, use data to show that low-noise surfaces are the most used intervention in urban contexts when screens or other solutions are not feasible, and validate their effectiveness with multi-year monitoring. Ohiduzzaman et al. [55] conduct a state-of-the-art review comparing propagation techniques based on the source and conclude that changing the type of surface is an effective and cost-efficient measure when rolling noise dominates; it also provides the methodological framework for measuring and verifying the benefit during operation.

Since the 1970s, different approaches to design, materials, and construction techniques aimed at improving the interaction between tires and the road have led various international administrations to develop a wide variety of pavements that minimize noise generation and amplification. As a result, many European countries have invested with considerable success in the construction of low-noise pavements as an effective noise reduction measure in new and existing road infrastructure [56].

Extensive research carried out by many countries in recent years has concluded that the porosity of the wearing course is the main cause of noise reduction resulting from tire-road interaction (see, for example, [49]). These studies also concluded that long-term porosity conservation depends on the performance of the materials used (e.g., grain size, binder type and percentage), traffic conditions (e.g., speed, vehicle types and percentages) and maintenance operations aimed at reducing or eliminating the clogging they suffer in dry and hot climates, as experienced in Spain. Researchers such as Ohiduzzaman et al. [55] have demonstrated that a network of interconnected pores acts as an absorbent, dissipating acoustic energy within the pavement and breaking the resonances of tire cavities. Therefore, although porosity enables noise generated by tire-pavement interaction to be absorbed, this pavement characteristic cannot be exploited as it increases the likelihood of clogging and obstruction.

Ling et al. [57] investigate the mechanisms of pneumatic-pavement noise generation (air pumping, block vibration, and resonances), measurement techniques, and the design of quiet pavements (texture, porosity, and rubber or porous mixtures). They conclude that optimizing the rolling surface to act at the source offers reductions of between 3 and 6 dB, with environmental and economic benefits, and provides design criteria (macrotexture and porosity) and in situ monitoring, making it the first line of mitigation when rolling noise predominates.

Low-noise pavements act at the source and can provide initial reductions of 4–6 dB (A) compared to conventional dense mixtures, with favorable cost-effectiveness when integrated into maintenance strategies [58]. However, their performance is sensitive to texture, effective porosity, and evolution (climate, traffic, clogging), which requires the selection of mixture families with a stable stone skeleton and rich mastic that keep a useful texture and a functional void network for as long as possible [59]. Bohatkiewicz et al. [48], study several low-noise wearing courses and compare their effect on noise in situ using the SPB method [44], in addition to characterizing their absorption. The study explicitly states that road noise depends on surface texture, and that proper design of the mixture, and its composition, allows acoustic performance to be anticipated from the project stage. When quiet surfaces are used (e.g., BBTM 8S), they report SPB reductions of 1.8 to 5.1 dB (at 50–80 km/h) compared to adjacent reference sections (SMA/conventional) at the exposure level and show high correlations (R^2^ ≈ 0.53–0.96) between absorption and SPB levels, which directly links the chosen texture/layer with the levels measured at the receiver. They conclude that selecting and monitoring the wearing course (texture and porosity) is a primary measure for reducing acoustic exposure in service.

Therefore, the design process for noise-reducing pavement involves the following steps: (a) Select the wearing course based on its useful texture (sufficient macrotexture and negative texture) and its porosity, which will be moderate or high depending on the objective and the environment; (b) Control the mean profile depth (MPD), an indicator of macrotexture with minimum thresholds (≥1.2–1.3 mm), and limit megatexture to avoid impacts; (c) Ensure a functional pore network (without excess), which improves absorption without compromising durability; (d) Use modified materials (e.g., rubber), which help build favorable textures and porosities and provide additional damping; (e) Maintenance should include preventive cleaning and selective sealing to maintain acoustic function and prevent clogging. Finally, in addition, wearing courses must be designed to withstand traffic loads, have adequate grip, good drainage capacity, and provide proper user comfort in order to ensure safety.

As already mentioned, intervention on the pavement is an extremely effective measure for mitigating noise. However, it is important to bear in mind that the response to treatment can vary significantly depending on the composition, porosity, and specific characteristics of each pavement. Table 1 shows the noise levels of some frequently used mixtures.

The data obtained show that SMA mixtures exhibit noise levels comparable to those of PA mixtures, despite their greater porosity and absorption capacity. However, SMA mixtures have been found to be more durable, as evidenced in the study by Campuzano [60]. This paper highlights the benefits of SMA mixtures in terms of noise reduction, which, combined with their outstanding mechanical performance, make them an ideal choice for use on road pavements. For this reason, mixtures such as SMA have experienced an increase in popularity as low-noise mixtures complemented by the numerous advantages they offer due to their composition.

## 4. SMA Mixtures

### Description

First released in Germany in the mid-20th century, the SMA has found wide practical use in Europe due to its high quality, durability, and damage resistance [61]. The SMA design concept relies on stone-on-stone contact to provide strength and a rich mortar binder to provide durability. These objectives are usually achieved with a gap-graded aggregate coupled with fiber and/or polymer-modified, and a high asphalt content matrix.

SMA has a stone skeleton of interlocking crushed rock coarse aggregate, comprising largely single sized stone of a size appropriate to the laying thickness and required surface texture. The high content of coarse aggregate ensures stone-to-stone contact and high resistance to rutting. The narrow grading of the aggregate skeleton leaves a relatively high void content between the aggregate particles which is partly filled with a binder rich mastic mortar. The aggregate grading is similar to that of porous asphalt, but with the voids filled with mortar. The mortar comprises crushed rock fine aggregate, filler, bitumen or modified bitumen and a stabilizing additive, generally fibers. The composition of the mortar is very important in determining the performance of the SMA; a very high binder content (typical binder content is 6–7%) is essential to ensure durability and laying characteristics. Sufficiently high binder contents cannot be achieved using unmodified or unsterilized bitumen without binder drainage; hence the need for a fiber stabilizer, or absorptive fillers or modified binders [62].

**Figure 4:**
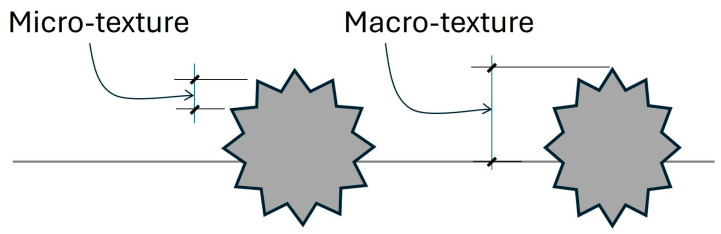
Microtexture and macrotexture.

Normally, SMA mixtures are primarily used on pavements carrying heavy traffic volumes, heavy loads and/or high tire pressures. The macrotexture of SMA (Figure 4) is usually superior to that of dense-graded asphalt, which reduces noise generation due to air pumping and block vibration in the high intermediate speed range. As mentioned earlier, the increase in service life and improved performance can offset the additional cost when using SMA in lieu of dense-graded asphalt.

Other potential uses of SMA include high-stress pavement areas, such as at intersections and truck terminals, and thin overlays, since a 5–8 mm nominal size SMA mix is well suited to providing thin overlays (20–30 mm) to renew frictional resistance or maintenance of existing pavements [63].

Vázquez et al. [64] conducted a study of two mixtures, SMA11 and SMA16, constructed in Spain with the same modified binder, evaluated in situ by measuring CPX at 50 and 80 km/h, texture (MPD, spectra), acoustic absorption in an impedance tube, and dynamic stiffness. They demonstrated that particle size distribution and texture spectrum condition the CPX level (SMA16, with greater texture at many wavelengths, has slightly higher levels than SMA11) and propose acoustic labeling to be applied to pavement management.

Gardziejczyk et al. [65] analyze low-noise SMA with rubber granules and several bitumen modifiers. Although the main focus is on viscoelasticity (4PB-PR stiffness and elasticity modulus), field tests are compiled and discussed that show that SMA LA (with 9–14% voids) achieves CPX reductions of between 2.5 and 4.0 dB compared to traditional solutions, and that SMA8 LA with rubber has higher elasticity than mixtures without rubber.

Vaitkus et al. [16] optimized wearing courses for noise reduction by comparing different SMA mixtures. They concluded that, at the cost of a certain mechanical penalty, noise levels could be reduced by between 3 and 5 dB compared to reference mixtures by increasing the void content.

Acoustic performance. International research shows that the initial reduction in noise levels achieved by porous and semi-porous mixtures (e.g., PA/ZOAB and variants) tends to decrease over time (between 0.2 and 0.3 dB per year in temperate climates) due to clogging and aging of the binder [66], while SMA retains texture integrity better as long as the aggregates, binder, and fibers are properly selected. In cold climates, long-term tests show behavior that depends on macrotexture and maintenance. In hot, dry climates, clogging is generally slower, and early stabilization may even slightly reduce CPX during the first few months.

Ohiduzzaman et al. [55] conducted a state-of-the-art review of road noise reduction and measurement techniques, comparing propagation solutions (screens) with source-based measures (quiet pavements, tires). They conclude that changing the type of surface, its texture, and porosity is an effective, repeatable, and cost-effective measure for reducing noise when the dominant component is rolling noise, and they support the use of CPX and SPB for verification and longitudinal monitoring.

In Spain, CPX and SPB campaigns have been carried out to classify and acoustically label asphalt mixtures, paying special attention to their correlation with texture and their temporal evolution. Recent demonstration projects have explored the incorporation of end-of-life tire powder (ELT) and plastic waste into SMA-type matrices to improve attenuation without compromising mechanical durability.

Regulations. From a normative perspective, in Europe, SMA are included in EN 13108-5 [67] and, in Spain, in its transposition UNE-EN 13108-5 [67], as well as in references and recommendations in specialized technical documents. Although their historical implementation in Spain has been more limited than in northern European countries, interest in their structural durability and acoustic benefits has grown over the last decade, with applications in the wearing courses of motorways, high-capacity roads, and urban environments where mitigation at noise source is very helpful [68].

In Spain, the PG-3 [69] is a comprehensive standard that governs the execution of state-administered highways, expressways, and motorways. Through OC 3/2019 [70], the Directorate General for Roads of the Spanish Ministry of Transport has created a new article (544) for PG-3, which includes SMA mixtures as a wearing course. This article defines the scope of application of SMA mixtures and their technical design and maintenance requirements (e.g., discontinuous particle size distribution, binder and stabilizing fiber content, void/volumetric limits, and performance verification tests during manufacturing and on site). According to PG-3, SMA has now become a preferred alternative to BBTM on sections with high traffic intensity, emphasizing its greater durability and good performance against crack reflection.

## 5. Comparative Study of SMA Mixtures

### 5.1. Need of the Study

Despite the accumulated knowledge, there is a lack of in situ longitudinal studies in hot and dry Mediterranean climates that comprehensively quantify, in real infrastructures, the role of macrotexture (MPD), in situ voids, and the percentage of rubber on the CPX and its evolution.

Licitra et al. [50] conducted multi-year monitoring of rubberized surfaces using CPX and proposed a multivariate regression model that incorporates traffic and weather conditions to estimate the evolution (dB/year) of L_CPX_. The authors explicitly emphasize that “more knowledge about long-term acoustic performance is required” in order to be able to plan implementation and maintenance.

Additionally, Sirin et al. [71] demonstrated, through a study conducted in Qatar, that ambient temperature significantly affects TPIN and that the temperature correction factors used in standards (obtained for a range of 5–35 °C) cannot be directly extrapolated to extremely hot climates, which creates uncertainty in longitudinal series. This highlights the methodological gap that exists in hot climates and the need to adjust models to infer “acoustic life” in these environments.

Similarly, Andriejauskas et al. [72] conducted a study in which they performed a spectral analysis of the ageing of low-noise mixtures. They concluded that rapid acoustic ageing is a critical problem and that a better understanding of its behavior is necessary to optimize designs for regions with severe climates. Although the study was conducted in a Baltic climate, the message can be extrapolated: climate determines acoustic durability even in warm climates, and, therefore, it is necessary to obtain more data series that allow reliable acoustic lifespans to be extrapolated.

In conclusion, traffic noise is one of the main sources of environmental disturbance in Europe. SMA with crushed aggregate, modified with scrap ELT or plastic waste have shown an initial reduction between 3 and 6 dB (A) without a significant reduction in mechanical response. However, there are only a few longitudinal studies that measure acoustic durability in situ, and none in the hot, dry climate of southern Spain.

### 5.2. Data

The LIFE-Soundless project [33], carried out by the Regional Ministry of Development, Infrastructure, and Land Planning of the Regional Government of Andalusia (2019), demonstrated the effectiveness and durability of SMA-type mixtures in mitigating noise emission at its source. It also focused on the effectiveness of these mixtures in Mediterranean climates (southern Europe), which are very different from those used in northern countries. Southern Europe has more experience in the use and performance of porous sound-absorbing mixtures, but in dry and warm climates, these types of porous mixtures have not yielded the expected results due to the clogging of voids or problems with aggregate detachment in braking areas under severe tangential stresses.

The project provides a database consisting of five CPX measurement campaigns on nine lanes with conventional SMA mixtures, as well as SMA mixtures modified with ELT powder and a reference mixture AC16 surf, a mixture widely used in the zone. Compared to a freshly laid reference mixture (AC16 surf), the tested SMA mixtures reduce road noise by 3 dB at low speeds (50 km/h) and by 4 dB at medium speeds (80 km/h). SMA8, which contains 1.5% rubber powder from ELT, is the quietest of all the asphalt mixes analyzed.

In addition, this solution has reduced the number of people affected by noise on the A-8058 highway by 50%, and by more than 20% on the A-376 highway. The estimated reduction in healthcare costs thanks to the improved acoustic performance of Soundless mixtures is very significant, guaranteeing the long-term viability of these asphalt mixtures.

For all these reasons, the project represents a unique opportunity, as it makes available to the public multiple CPX campaigns on nine lanes with SMA/XSMA formulations and dense monitoring. This article uses this database to: (i) develop a model for CPX as a function of MPD, voids, rubber percentage, time and temperature; (ii) estimate acoustic lifespans according to a criterion of increase < 3 dB; and (iii) suggest design thresholds for MPD and voids that could be incorporated, after further studies, into Spanish standards. It is expected that the findings of this study will contribute to the widespread use of low-noise, durable and sustainable pavements in Spain.

The measurement criteria defined in Section 2.2 have been used.

#### 5.2.1. Data Filtering

The standard includes corrections for comparison between campaigns and devices: (a) Speed: normalization to a reference speed of 50/80 km/h, although other ranges may be established; (b) Temperature: thermal influence (air, tire, surface) is corrected to 20 °C using coefficients defined in ISO/TS 13471-1. This standard states that, in CPX, the operating range (≈5–35 °C) can introduce substantial level changes if not normalized. (c) Tire: correction for tire hardness and condition and pressure/load requirements. (d) Device: correction of the enclosure by frequency band to compensate for reflections and geometric differences between enclosures.

The L_CPX_ result for a lane is obtained by energetically averaging positions 1 and 2 (mandatory) and, if applicable, averaging valid passes; then the tires are combined for L_CPX:P_, L_CPX:H_, or a combined index. The CPX values from six campaigns (months 0, 3, 6, 12, 18, 24) were extracted from the tables of the Life Soundless project (A-8058 and A-376; lanes and SMA/XSMA mixtures and AC16 reference). Ambient temperatures (22–26 °C) were associated with each campaign to allow for thermal correction. For the study, SMA 8 mixtures with variations in their composition were analyzed, which were laid on the A-8058 and A-376 highways as part of the Life Soundless Project.

The mixtures analyzed are described in Table 2:

#### 5.2.2. Data Used for the Study

**Figure 5:**
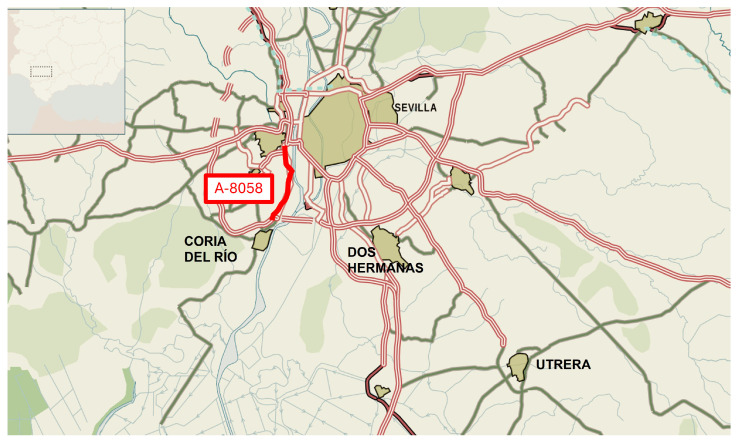
Scenario 1. A-8058 road between Seville and Coria. Location within the road network.

**Figure 6:**
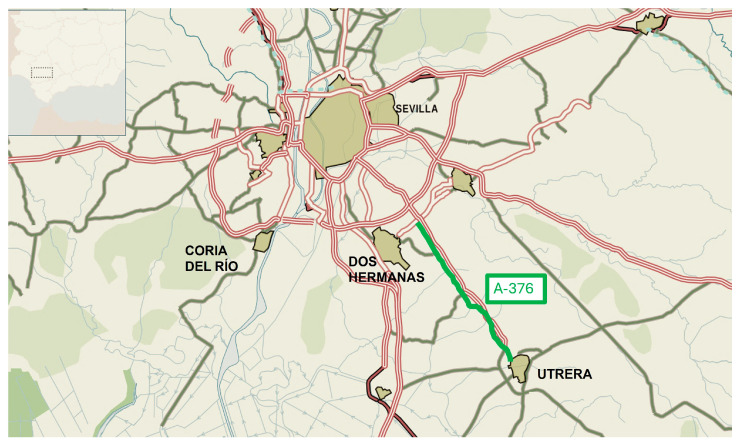
Scenario 2. A-376 road between Seville and Utrera. Location within the road network.

The roads included in the study were:

- Scenario 1: A-8058 between Seville and Coria (Figure 5). SMA mixtures were modified with end-of-life tires (ELTs), laid in different proportions.
- Scenario 2: A-376 between Seville and Utrera (Figure 6). In this case, the mixtures were modified with plastic, nylon and ELTs in different proportions. The AC-16 surf mixture reference was also laid.

**Figure 7:**
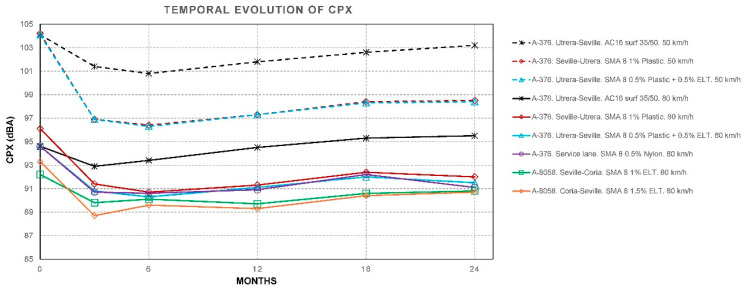
Temporal evolution of CPX.

**Figure 8:**
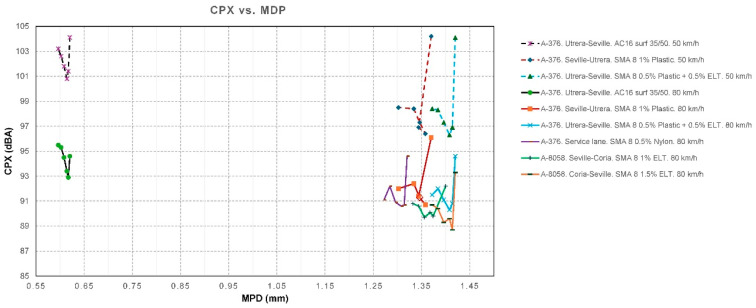
CPX vs. MPD. Data for all mixtures.

**Figure 9:**
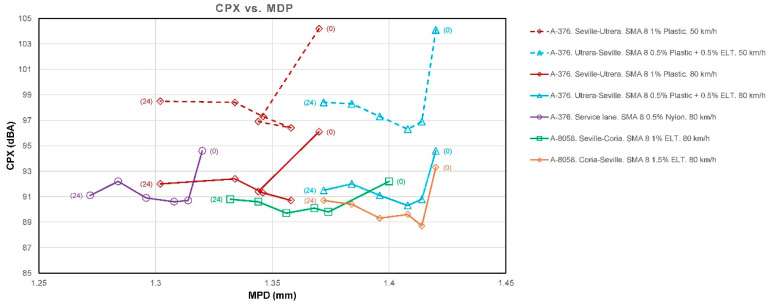
CPX vs. MPD. Data for SMA mixtures. Months in brackets.

The CPX noise measurement campaigns were carried out at 50 and 80 km/h at the start (month 0) and at 3, 6, 12, 18, and 24 months using an ISO 11819-2 trailer [43], with the results shown in Table 3, Table 4, Table 5, Table 6, Table 7, Table 8 and Table 9. Figure 7 shows the temporal evolution of CPX. Figure 8 shows the values of CPX vs. MPD for all the mixtures, while Figure 9 shows the values for SMA mixtures only.

### 5.3. Analysis Methodology

The methodology followed in the study aims to quantify how CPX (dB (A)) varies as a function of MPD, amount of voids, percentage of additives, age (months), and temperature per lane and season.

The variables used were (See Table 3, Table 4, Table 5, Table 6, Table 7, Table 8 and Table 9):

- CPX response variable (dB (A)): level measured per campaign and lane. It is denoted as CPXij, where i is the campaign (0, 3, 6, 12, 18, or 24 months) and j is the section/lane.
- Predictors (all at section and campaign level):
  ○ MPD [mm]: average depth of macrotexture.
  ○ Voids [%]: in situ porosity assigned by mix family.
  ○ Additives [%]: percentage of modification with ELT powder and/or plastic (0; 0.5; 1; 1.5). Modelled as a continuous variable to estimate the marginal effect per percentage point.
  ○ Months [month]: age since in-service (0–24). Includes polishing/pore cleaning/early stabilization processes.
  ○ Tair [°C]: ambient temperature during auscultation (22–26 °C).

#### Statistical Model

To adjust the data set indicated in the previous point, a linear regression analysis was performed using the least squares method. This analysis shows how a straight line defines the evolution of the CPX based on the selected variables, and the effect of each variable on the final value.

The proposed equation would be of the following type (1):

(1) $$\begin{array}{c} {CPX}_{ij}=\beta_{0}+\beta_{1}\cdot {MPD}_{ij}+\beta_{2}\cdot {Voids}_{ij}+\beta_{3}\cdot {Additives}_{ij}+\;\beta_{4}\\ \cdot \;{Months}_{ij}+\;\beta_{5}\cdot {Tair}_{ij} \end{array}$$

where *β*_0_ is a constant and the coefficients *β*_1_ to *β*_5_ correspond to the average change in CPX (dBA) due to 1 mm additional MPD, 1% of voids, 1% of additives, 1 month of service and a 1 °C increase in air temperature, respectively.

The results obtained from the model show:

- The factor for MDP is *β_1_* = −2.437 dB/mm. This means that, provided all other factors remain constant, a higher MPD indicates a lower CPX. In a typical range, macrotexture values are between 0.6 and 1.4 mm. Moving from the lower to the upper end of this range implies a difference of 0.8 mm, which, according to the model, represents a decrease of 0.8·(−2.437) =−1.95 dB. This is consistent with the physical hypothesis: an “optimal” macrotexture reduces air pumping and tire block excitation in the range where TPIN dominates.
- A high correlation between macrotexture values and the void index has been observed, and therefore, only one of them should be considered to draw more accurate conclusions and avoid collinearity between variables. In the developed model, it is macrotexture (rather than void content) the variable that defines CPX.
- The factor for additives is *β*_3_ = −1177 dB per 1%. This result indicates that adding 1.0% additives reduces the noise level by approximately 1.18 dB compared to the absence of rubber. This data provides us with one of the first conclusions of the study: the clear acoustic benefit of adding rubber or plastic to this type of mixture.
- *β*_4_ = −0.0329 dB/month. This value, approximately −0.40 dB per year, reflects the shape of the observed data graphs (Figure 7), which show a decline during the first few months, followed by stabilization. However, while this result is reasonable for these time intervals, extrapolating it to many years is not recommended.
- *β*_5_ = −0.123 dB/°C. At higher air temperatures, slightly less noise is produced, with an approximate reduction of 1.23 dB for every 10 °C increase. This is because the rubber and binder become softer and reduce some of the vibration.

The equation resulting from the model is as follows (2):

(2) $$\begin{array}{c} {CPX}_{ij}=99.084-2.347\cdot {MPD}_{ij}-1.177\cdot {Additives}_{ij}-0.0329\cdot \;\beta_{4}\cdot \\ {Months}_{ij}-0.1234\cdot {Tair}_{ij}+\;\varepsilon_{ij} \end{array}$$

The values obtained for R^2^ and adjusted R^2^ (0.415 and 0.339, respectively) indicate that the model explains between 33% and 41% of the variability in CPX. While the model captures the main physical trends, it leaves appreciable residual variance (unmodeled factors).

These values are consistent with those obtained in in situ pavement studies, in which environmental and operational variability introduces some background noise. The direction of the estimated effects was consistent with theory (MPD, additives percentage, temperature and time), although a significant portion of unexplained variance remains, attributable to unmodeled factors (tire type, microtexture, accurate operating regime, traffic pattern, punctual humidity, etc.). For practical purposes, the obtained equation is suitable for comparative and sensitivity analyses within the data domain (MPD ≈ 0.6–1.4 mm; additives 0–1.5%; 0–24 months; Tair between 22–26 °C), while the punctual prediction must be accompanied by uncertainty intervals (typical error ≈ ±1.6 dB).

**Figure 10:**
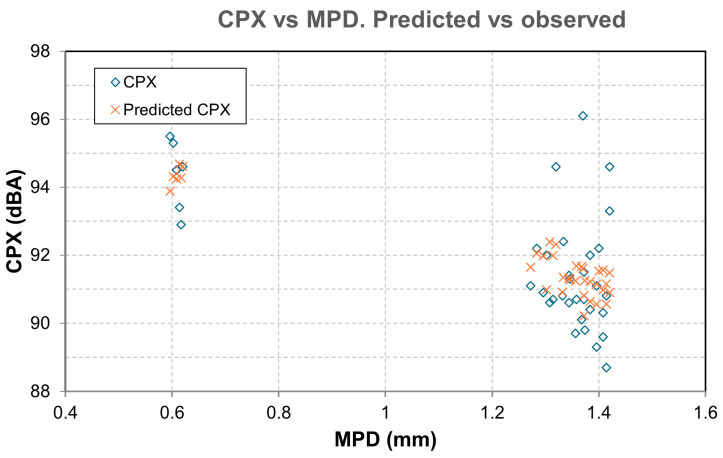
Observed vs. predicted. CPX values vs. MPD.

**Figure 11:**
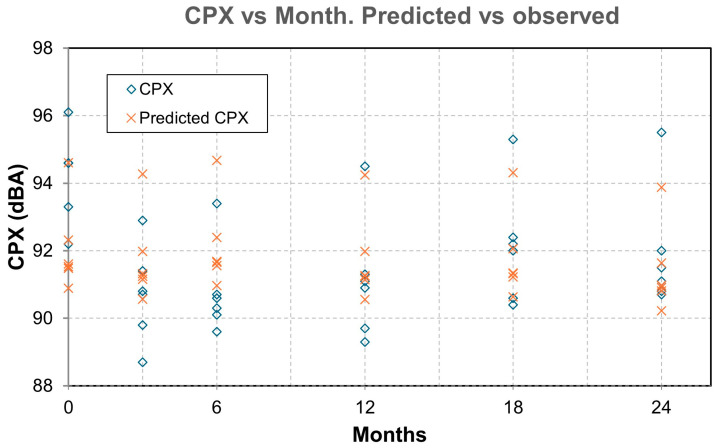
Observed vs. predicted. CPX values vs. month.

As initial results (Figure 10 and Figure 11), we can conclude that there is a clear trend: the greater the texture depth, the lower the CPX (except in the AC16 reference), and that most SMA fluctuates ±2 dB in 24 months, while AC16 increases by more than 2 dB.

## 6. Discussion

The initial improvement of 4–6 dB and the slight improvement observed in the first few months are consistent with studies conducted in Central Europe, Poland [73] and Denmark [74], despite the significant differences in climatic conditions between these regions and southern Spain. The hot, dry climate in Andalusia causes gaps in the pavement to become clogged within the first two years.

In order to guarantee a reduction of more than 4 dB for at least two years, it is recommended that an MPD of at least 1.25 mm and a void content of at least 11% to be required upon completion of the work. This extends OC 3/2019 [70], which currently imposes no acoustic limits.

The results confirm three main ideas:

- SMAs provide substantial initial noise reduction compared to dense mixes (AC),
- Surface parameters, such as macrotexture (MPD) and voids, are determining factors.
- Within two years, the CPX does not worsen. It even improves slightly in hot and dry climates. These trends are consistent with European experience: the initial advantage of low-noise pavements (3–6 dB) is well documented, although the magnitude and persistence depend on the type of mixture and the climate. In the Netherlands and Denmark, porous asphalt starts at –4 to –6 dB and shows annual losses of ~0.2–0.33 dB per year. Less porous materials, such as SMA, tend to stabilize their response provided there is no severe clogging [75,76].

### 6.1. Analysis Methodology MPD and Voids

The model relates MPD and porosity to the expected physical effects (the higher the MPD and porosity, the lower the CPX), although the statistical significance of this relationship is reduced due to low variability between measurement locations and collinearity with age (MPD decreases slightly over time). Sandberg [17] attributes the effect of MPD to reduced air pumping and block vibration within speed ranges where TPIN dominates, minimizing tire excitation. Values of MPD ≥ 1.25 mm should be recommended, as this is consistent with the observed data ranges and Danish reports on texture for low noise and low rolling resistance.

With respect to voids, evidence, especially in PA pavements, indicates that effective porosity facilitates absorption and attenuates Helmholtz cavity resonances. Ohiduzzaman et al. [55] point out that an excess of voids penalizes durability and accelerates the loss of benefits. SMA mixtures were designed with moderate voids (10–12%), a compromise that explains their acoustic stability compared to very open PA pavements.

### 6.2. Effect of Additives

The additives factor, β_3_, is −1.177 dB per 1%. This value does not represent a literal linear relationship across the entire range of results; rather, it demonstrates how additives provide a useful texture and elastic network in the mastic, as well as providing connected-void gradings without the need for extreme porosities. Vázquez et al. [64] concluded that SMA+ELT achieves lower CPX levels than conventional SMA and AC, which is consistent with this effect. In summary, additives contribute to acoustic benefits both superficially, due to texture and porosity, as well as through the vibroacoustic properties of the compound.

### 6.3. Temporal Evolution of CPX

According to the obtained results (β_4_ = −0.0329 dB/month ≃ −0.40 dB/year), the CPX decreases slightly during the first two years. This is consistent with the early stabilization phase observed in hot and dry climates, during which natural cleaning of fine particles and controlled polishing of micro-roughness reduce certain spectral contributions without yet inducing clogging or ageing processes that increase noise. While the specialized literature does show growth of between 0.2 and 0.33 dB per year in the case of highly porous mixtures, in the case of SMA with moderate porosity and a good stone skeleton, behavior may remain flat or decrease slightly in the short term. This nuance is important when drafting technical specifications for construction projects involving open solutions, as it highlights the need to specify the texture and voids required for the finished work and maintenance (cleaning).

### 6.4. Temperature

The air temperature factor, β_5_ = −0.123 dB/°C, partly reflects non-parametric thermal corrections and unmodeled covariates, such as time of day, binder stiffness, and tire conditions. Normalization to 20 °C is established in ISO/TS 13471-1 [77], precisely because CPX is sensitive to thermal variations in the tire, air, and surface. Therefore, it seems advisable to report normalized CPX and keep complete thermal records in future campaigns to better isolate this effect, even though its influence on the results is small.

### 6.5. Design Implications for Spain and Incorporation into PG-3

OC 3/2019 [70] incorporates SMA mixtures into the Spanish framework. The sector’s experience [78] suggests that SMA mixtures have high durability and low noise levels when the mastic is controlled at 6–7% and stabilized with fibers. These results support requiring mandatory MPD thresholds of ≥1.25 mm and void thresholds of ≥11% for SMA upon completion of work in Mediterranean climates. The results also support the feasibility of requiring CPX verification during operation at 50 and 80 km/h. As an operational criterion, we propose setting an acoustic life threshold of increase < 3 dB, consistent with CEDR guidelines for performance throughout the life cycle.

### 6.6. Future Works

The adjustment was made with 36 observations, so conducting an experimental study with more sections and greater variability would improve the inference obtained, allowing for a more rigorous adjustment of the model. In addition, the study obtained a reasonable adjustment, but it is advisable to refine the model with nonlinear terms and interactions MPD-Months or Voids-Rubber.

## 7. Conclusions

In this paper, data from the Soundless and other projects have been collected and a comparative study on the noise produced by different SMA mixtures with different additives, such as rubber crumb and polymeric additives, has been carried out, in order to analyze the noise reduction in each of them with respect to a standard mixture. Besides the considerations made in Section 5, the main conclusions drawn from the study are the following:

- SMA mixtures containing rubber reduce CPX by 4–6 dB compared to dense AC16. This is consistent with the results from the Life Soundless project.
- Of all the analyzed variables, the percentage of rubber is one of the most significant. According to the analysis, increasing the rubber content by 1% reduces CPX by 1.18 decibels, approximately.
- In terms of construction time, none of the analyzed sections show increases greater than 3 dB in 24 months, so it can be stated that the minimum acoustic life exceeds three years.
- The authors recommend further studies to confirm that, upon completion of works in Mediterranean climates, the MPD value should exceed 1.25 mm and the void percentage should exceed 11%. These studies should be a part of more comprehensive research on SMA, with the aim of defining the parameters of these mixtures to be eventually included in Spanish regulations.

## Figures and Tables

**Table 1:** Asphalt mixtures and their noise levels.

Asphalt Mixture	Noise Level dB(A)	Source
SMA11 (Spain)	95.1	[21]
SMA11S (Czechia)	98.2	[16]
EACC 8 mm (exposed aggregate concrete)	98.0	[16]
Double-layer porous asphalt (DPA)	≈93.0	[28]

**Table 2:** Asphalt mixtures analyzed with their initial macrotexture and % air voids.

Mixture	MPD_0_ (mm)	Air Voids (%)
SMA 8 1% ELT	1.35	12.5
SMA 8 1.5% ELT	1.35	10.0
SMA 8 0.5% Nylon	1.20	8.0
SMA 8 1% Plastic	1.35	10.0
SMA 8 0.5% Plastic + 0.5% ELT	1.35	11.5
AC16 surf 35/50	0.80	3.0

**Table 3:** A-376 Seville-Utrera. 50 km/h.

Lane	Speed	Mixture	CPX	MPD	% Additive	Months	Air Temp.
A-376 Seville-Utrera	50	SMA 8 1% Plastic	104.2	1.37	1	0	24
A-376 Seville-Utrera	50	SMA 8 1% Plastic	96.9	1.344	1	3	26
A-376 Seville-Utrera	50	SMA 8 1% Plastic	96.4	1.358	1	6	22
A-376 Seville-Utrera	50	SMA 8 1% Plastic	97.3	1.346	1	12	24
A-376 Seville-Utrera	50	SMA 8 1% Plastic	98.4	1.334	1	18	22
A-376 Seville-Utrera	50	SMA 8 1% Plastic	98.5	1.302	1	24	24

**Table 4:** A-376 Utrera-Seville. 50 km/h.

Lane	Speed	Mixture	CPX	MPD	% Additive	Months	Air Temp.
A-376 Utrera-Seville	50	SMA 8 0.5% Plastic + 0.5% ELT	104.1	1.42	1	0	24
A-376 Utrera-Seville	50	SMA 8 0.5% Plastic + 0.5% ELT	96.9	1.414	1	3	26
A-376 Utrera-Seville	50	SMA 8 0.5% Plastic + 0.5% ELT	96.3	1.408	1	6	22
A-376 Utrera-Seville	50	SMA 8 0.5% Plastic + 0.5% ELT	97.3	1.396	1	12	24
A-376 Utrera-Seville	50	SMA 8 0.5% Plastic + 0.5% ELT	98.3	1.384	1	18	22
A-376 Utrera-Seville	50	SMA 8 0.5% Plastic + 0.5% ELT	98.4	1.372	1	24	24
A-376 Utrera-Sevilla	50	AC16 surf 35/50	104.1	0.62	0	0	24
A-376 Utrera-Sevilla	50	AC16 surf 35/50	101.4	0.617	0	3	26
A-376 Utrera-Sevilla	50	AC16 surf 35/50	100.8	0.614	0	6	22
A-376 Utrera-Sevilla	50	AC16 surf 35/50	101.8	0.608	0	12	24
A-376 Utrera-Sevilla	50	AC16 surf 35/50	102.6	0.602	0	18	22
A-376 Utrera-Sevilla	50	AC16 surf 35/50	103.2	0.596	0	24	24

**Table 5:** A-376 Seville-Utrera. 80 km/h.

Lane	Speed	Mixture	CPX	MPD	% Additive	Months	Air Temp.
A-376 Seville-Utrera	80	SMA 8 1% Plastic	96.1	1.37	1	0	24
A-376 Seville-Utrera	80	SMA 8 1% Plastic	91.4	1.344	1	3	26
A-376 Seville-Utrera	80	SMA 8 1% Plastic	90.7	1.358	1	6	22
A-376 Seville-Utrera	80	SMA 8 1% Plastic	91.3	1.346	1	12	24
A-376 Seville-Utrera	80	SMA 8 1% Plastic	92.4	1.334	1	18	22
A-376 Seville-Utrera	80	SMA 8 1% Plastic	92	1.302	1	24	24

**Table 6:** A-376 Utrera-Seville. 80 km/h.

Lane	Speed	Mixture	CPX	MPD	% Additive	Months	Air Temp.
A-376 Utrera-Seville	80	SMA 8 0.5% Plastic + 0.5% ELT	94.6	1.42	1	0	24
A-376 Utrera-Seville	80	SMA 8 0.5% Plastic + 0.5% ELT	90.8	1.414	1	3	26
A-376 Utrera-Seville	80	SMA 8 0.5% Plastic + 0.5% ELT	90.3	1.408	1	6	22
A-376 Utrera-Seville	80	SMA 8 0.5% Plastic + 0.5% ELT	91.1	1.396	1	12	24
A-376 Utrera-Seville	80	SMA 8 0.5% Plastic + 0.5% ELT	92	1.384	1	18	22
A-376 Utrera-Seville	80	SMA 8 0.5% Plastic + 0.5% ELT	91.5	1.372	1	24	24
A-376 Utrera-Seville	80	AC16 surf 35/50	94.6	0.62	0	0	24
A-376 Utrera-Seville	80	AC16 surf 35/50	92.9	0.617	0	3	22
A-376 Utrera-Seville	80	AC16 surf 35/50	93.4	0.614	0	6	22
A-376 Utrera-Seville	80	AC16 surf 35/50	94.5	0.608	0	12	24
A-376 Utrera-Seville	80	AC16 surf 35/50	95.3	0.602	0	18	22
A-376 Utrera-Seville	80	AC16 surf 35/50	95.5	0.596	0	24	24

**Table 7:** A-376. Service Line. 80 km/h.

Lane	Speed	Mixture	CPX	MPD	% Additive	Months	Air Temp.
A-376 Service Lane	80	SMA 8 0.5% Nylon	94.6	1.32	0.5	0	22
A-376 Service Lane	80	SMA 8 0.5% Nylon	90.7	1.314	0.5	3	26
A-376 Service Lane	80	SMA 8 0.5% Nylon	90.6	1.308	0.5	6	22
A-376 Service Lane	80	SMA 8 0.5% Nylon	92.2	1.284	0.5	18	22
A-376 Service Lane	80	SMA 8 0.5% Nylon	90.9	1.296	0.5	12	24
A-376 Service Lane	80	SMA 8 0.5% Nylon	91.1	1.272	0.5	24	24

**Table 8:** A-8058 Seville-Coria. 80 km/h.

Lane	Speed	Mixture	CPX	MPD	% Aditive	Months	Air Temp.
A-8058 Seville-Coria	80	SMA 8 1% ELT	92.2	1.4	1	0	22
A-8058 Seville-Coria	80	SMA 8 1% ELT	89.8	1.374	1	3	26
A-8058 Seville-Coria	80	SMA 8 1% ELT	90.1	1.368	1	6	22
A-8058 Seville-Coria	80	SMA 8 1% ELT	89.7	1.356	1	12	24
A-8058 Seville-Coria	80	SMA 8 1% ELT	90.6	1.344	1	18	22
A-8058 Seville-Coria	80	SMA 8 1% ELT	90.8	1.332	1	24	24

**Table 9:** A-8058 Coria-Seville. 80 km/h.

Lane	Speed	Mixture	CPX	MPD	% Aditive	Months	Air Temp.
A-8058 Coria-Seville	80	SMA 8 1.5% ELT	93.3	1.42	1.5	0	24
A-8058 Coria-Seville	80	SMA 8 1.5% ELT	88.7	1.414	1.5	3	26
A-8058 Coria-Seville	80	SMA 8 1.5% ELT	89.6	1.408	1.5	6	22
A-8058 Coria-Seville	80	SMA 8 1.5% ELT	89.3	1.396	1.5	12	24
A-8058 Coria-Seville	80	SMA 8 1.5% ELT	90.4	1.384	1.5	18	22
A-8058 Coria-Seville	80	SMA 8 1.5% ELT	90.7	1.372	1.5	24	24

## Data Availability

The original contributions presented in this study are included in the article. Further inquiries can be directed to the corresponding author.

## Abbreviations

The following abbreviations are used in this manuscript:

ADOT	Arizona Department of Transportation
AVAS	Acoustic Vehicle Alerting System
BBTM	Very Thin Bituminous Concrete (Béton Bitumineux Très Mince)
CEDEX	Centro de Estudios y Experimentación de Obras Públicas (Spain) Centre for Studies and Experimentation of Public Works (Spain)
CEDR	Conference of European Directors of Road
CPX	Close Proximity
EU	European Union
ELT	End-of-life tire
FHWA	Federal Highway Administration
MFOM	Ministerio de Transportes y Movilidad Sostenible (Spain) Spanish Ministry of Transport and Sustainable Mobility
MPD	Mean Profile Depth
OECD	Organization for Economic Co-operation and Development
PA	Porous Asphalt
PG-3	General Technical Specifications for Road and Bridge Works. (Spain)
RD	Royal Decree
SMA	Stone Mastic Asphalt, also known as Stone Matrix Asphalt
SPB	Statistical Pass By
TPIN	Tire-pavement interaction noise
WHO	World Health Organization.
ZOAB	Dutch acronym for porous asphalt
